# Drp1-mediated mitochondrial fission regulates calcium and F-actin dynamics during wound healing

**DOI:** 10.1242/bio.048629

**Published:** 2020-05-04

**Authors:** Susana Ponte, Lara Carvalho, Maria Gagliardi, Isabel Campos, Paulo J. Oliveira, António Jacinto

**Affiliations:** 1 CEDOC, Chronic Diseases Research Center, NOVA Medical School/Faculdade de Ciências Médicas, Universidade NOVA de Lisboa, 1169-056 Lisboa, Portugal; 2 Animal Platforms, Champalimaud Centre for the Unknown, 1400-038 Lisboa, Portugal; 3 CNC, Center for Neuroscience and Cell Biology, University of Coimbra, UC Biotech Building, 3060-197 Cantanhede, Portugal

**Keywords:** Drp1, F-actin, Calcium, Mitochondria, Mitochondrial dynamics, Wound healing

## Abstract

Mitochondria adapt to cellular needs by changes in morphology through fusion and fission events, referred to as mitochondrial dynamics. Mitochondrial function and morphology are intimately connected and the dysregulation of mitochondrial dynamics is linked to several human diseases. In this work, we investigated the role of mitochondrial dynamics in wound healing in the *Drosophila* embryonic epidermis. Mutants for mitochondrial fusion and fission proteins fail to close their wounds, indicating that the regulation of mitochondrial dynamics is required for wound healing. By live-imaging, we found that loss of function of the mitochondrial fission protein Dynamin-related protein 1 (Drp1) compromises the increase of cytosolic and mitochondrial calcium upon wounding and leads to reduced reactive oxygen species (ROS) production and F-actin defects at the wound edge, culminating in wound healing impairment. Our results highlight a new role for mitochondrial dynamics in the regulation of calcium, ROS and F-actin during epithelial repair.

## Abstract (precis)

**Summary:** We show that mitochondrial dynamics proteins are required for epithelial repair. Drp1 loss-of-function leads to defects in the dynamics of cytosolic and mitochondrial calcium, ROS production and F-actin upon wounding.

## INTRODUCTION

Mitochondria perform critical cellular functions such as energy production, regulation of calcium (Ca^2+^), redox homeostasis and cell death (El-Hattab and Scaglia, 2016). Mitochondrial shape is controlled by antagonizing fusion and fission events (Lewis and Lewis, 1914; Nunnari et al., 1997), described as mitochondrial dynamics, which allow mitochondria to adapt to cellular demands (Nunnari and Suomalainen, 2012).

Dynamin-related proteins regulate mitochondrial dynamics through their GTPase activity (Hoppins et al., 2007). Mitochondrial fission is accomplished by Dynamin-related protein 1 (Drp1). Upon activation, Drp1 is recruited from the cytosol to the mitochondria, oligomerizes and constricts this organelle until its division is achieved (Bleazard et al., 1999; Labrousse et al., 1999; Smirnova et al., 2001; Yoon et al., 2001). Mitochondrial fusion requires the merging of both the outer (OMM) and the inner mitochondrial membranes (IMM). Mitofusin 1 (Mfn1) and Mitofusin 2 (Mfn2) are responsible for OMM fusion (Rojo et al., 2002), while Optic atrophy 1 (Opa1) mediates fusion of the IMM (Griparic et al., 2004; Olichon et al., 2003).

Regulation of mitochondrial dynamics is essential for development (Chen et al., 2003; Ishihara et al., 2009; Waterham et al., 2007) and dysregulation of its machinery is implicated in a wide range of human diseases, including neuropathies, type II diabetes and cancer (Anderson et al., 2018; Ranieri et al., 2013; Rovira-Llopis et al., 2017). However, the role of mitochondrial dynamics in other contexts, such as epithelial repair, is still largely unknown.

Wound healing in simple epithelia is characterized by the accumulation of F-actin and non-muscle myosin II (myosin) at the cell boundaries that face the wound, forming an actomyosin cable that contracts and brings cells together, thereby closing the hole (Bement et al., 1999; Danjo and Gipson, 1998; Kiehart et al., 2000; Xu and Chisholm, 2011). Additionally, wound healing involves cell crawling mediated by actin protrusions (Abreu-Blanco et al., 2012a; Verboon and Parkhurst, 2015) and cellular rearrangements (Carvalho et al., 2018; Razzell et al., 2014).

Recent studies suggest that mitochondria might be required for tissue repair by producing reactive oxygen species (ROS). They have shown that mitochondrial ROS promote wound healing by regulating F-actin and myosin at the wound edge, either by acting on Rho GTPases (Muliyil and Narasimha, 2014; Xu and Chisholm, 2014) or on cell­–cell junction remodelling (Hunter et al., 2018). In this work, we show that the mitochondrial dynamics machinery is essential for repair, as mutants for these proteins fail to close epithelial wounds. In particular, the fission protein Drp1 is required for F-actin accumulation at the wound edge, for proper cytosolic and mitochondrial Ca^2+^ dynamics and for ROS production upon wounding. Our work reveals a novel role for mitochondrial fission in regulating ROS, Ca^2+^ and F-actin dynamics during epithelial repair.

## RESULTS

### Mitochondrial dynamics proteins are required for wound healing

**Fig. 1.**
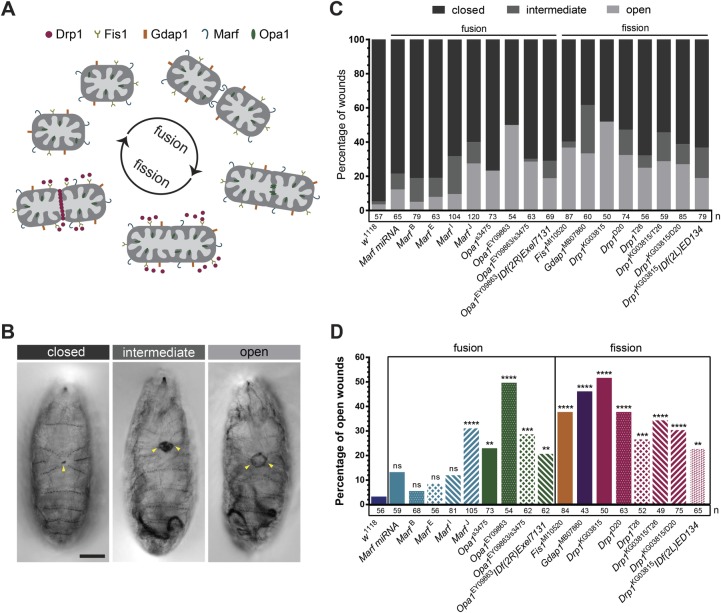
**Mitochondrial dynamics proteins are required for wound healing.** (A) Scheme of the proteins involved in mitochondrial dynamics used in the wounding assay screen. (B) Representative images of hatching larvae, 16 h after wounding, showing the three observed wound phenotypes: closed, intermediate and open. Closed wounds present a small scab, while open wounds show a ring of melanization around the hole. Intermediate wounds have more melanization than closed and open wounds but not a clear hole. Arrowheads point to the wound. Scale bar: 200 µm. (C) Graph of percentage of closed, intermediate and open wounds in controls (*w*^1118^) and mutant alleles for mitochondrial dynamics proteins. (D) Graph of percentage of open wounds in controls and mutant alleles for mitochondrial dynamics proteins. Regarding fusion, all *Opa1* alleles and heteroallelic combinations showed increased percentage of open wounds compared to controls; for *Marf*, only the *Marf*^J^ mutation shows significantly increased percentage of open wounds compared to controls. All the tested fission genes and heteroallelic combinations showed higher percentage of open wounds compared to controls. Fisher's exact test was used to test for significant differences between groups. *UAS-Marf miRNAi* was expressed under the control of the *da-Gal4* driver. The graph in D shows the same embryos from C, excluding those with intermediate wounds. ns, not significant (*P*>0.05), **P≤0.01, ***P≤0.001, ****P<0.0001. The number of embryos for each condition is shown below the bars in C and D.

To test whether the mitochondrial dynamics machinery (Fig. 1A) is required for epithelial repair, we performed a previously described wounding assay in the *Drosophila* embryonic epidermis (Campos et al., 2010). We laser-wounded late-stage embryos bearing wild-type and mutant alleles of mitochondrial dynamics proteins and assessed the wound-healing phenotype by the percentage of non-healing wounds.

Fig. 1A shows a scheme of mitochondrial dynamics with all the tested proteins represented. Regarding fusion, we tested four mutant alleles and one miRNA for Mitochondrial assembly regulatory factor (*Marf*, a *Drosophila* Mfn homolog); as well as two *Opa1* mutant alleles and two heteroallelic combinations. Concerning mitochondrial fission, we tested three *Drp1* alleles and three heteroallelic combinations. We also tested other fission regulators: Fission protein 1 (Fis1), which acts as a receptor for Drp1 at the OMM (Losón et al., 2013), and Ganglioside-induced differentiation associated protein 1 (GDAP1), whose function is not well understood (Huber et al., 2013).

We observed three types of wound closure phenotypes: open, intermediate and closed wounds (Fig. 1B). Closed wounds are identifiable by a small melanized spot. Open wounds show a melanized ring around the hole. In the intermediate phenotype, melanization occurs in a large circular area but a clear hole is absent, making it uncertain whether the wound is open or closed. Control embryos (*w*^1118^) have an outstanding capacity of epithelial repair, as 94.7% of the wounds are closed (Fig. 1C). Mutations in either mitochondrial fission or fusion genes increased the frequency of open and intermediate wounds (Fig. 1C).

As it is unclear whether the intermediate wounds represent a closure impairment or just a melanization defect, we excluded these wounds from the statistical analysis of the wound healing phenotype. Comparing only closed versus open wounds, all mitochondrial fission mutants showed higher percentage of open wounds than controls (Fig. 1D). Regarding mitochondrial fusion, from the four tested *Marf* alleles and the miRNA, only *Marf*^J^ showed an increased percentage of open wounds compared to controls. *Opa1* mutants showed a significant wound closure phenotype (Fig. 1D).

As we observed wound-closure defects for mutated versions of both fusion and fission proteins, these data suggest that the regulation of mitochondrial dynamics is necessary for wound healing.

### *Drp1* mutants show delayed wound healing

Mitochondrial fission mutants showed a more consistent wound healing phenotype than fusion mutants. Therefore, we decided to explore the role of mitochondrial fission in epithelial repair by focusing on the function of Drp1.

**Fig. 2.**
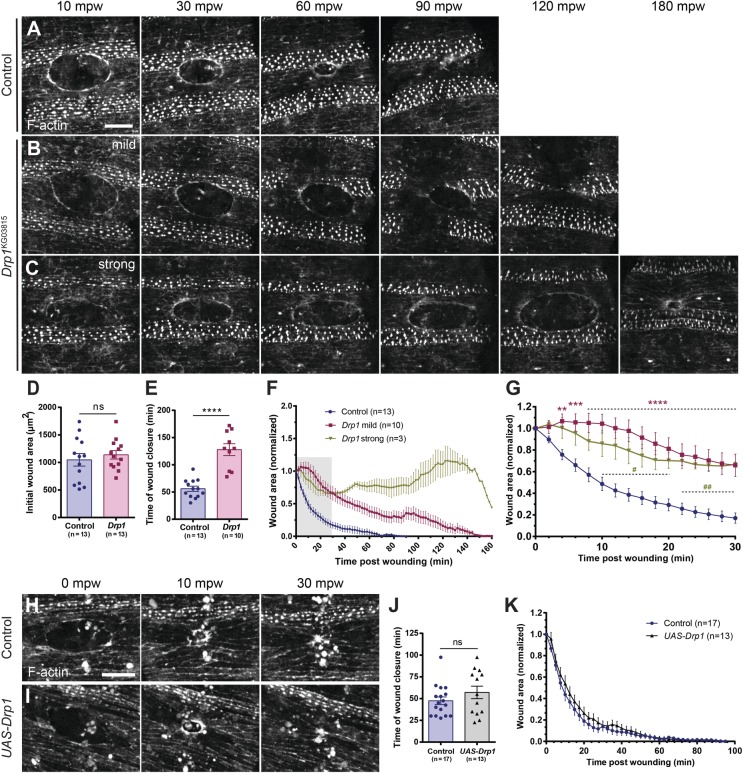
***Drp1* embryos show delayed wound healing.** (A–C) Maximum Z projections of the epidermis of control (A), *Drp1* mild (B) and *Drp1* strong (C) mutant embryos expressing an F-actin marker (*GFP::Moesin*) during wound closure. In *Drp1* mild mutants (B) wounds close slower than in controls (compare B with A). In *Drp1* strong mutants (C), although the wound contracts in the first 30–40 mpw, it then starts to expand (see 60–120 mpw). Later on, the wound contracts again and by 180 mpw it is almost closed. (D) Graph of average initial wound area in control and *Drp1* mutant embryos (strong and mild). (E) Graph of wound closure time in control and *Drp1* mutant embryos. Although the initial wound area of control and *Drp1* mutants is similar (D), *Drp1* mutants take longer to close their wounds (E). Unpaired *t*-test with Welch's correction was performed to test for significant differences between groups in D and E. ns, not significant (*P*>0.05), ****P≤0.0001. (F) Graph of average wound area in control, *Drp1* mild and *Drp1* strong mutants over time. *Drp1* mild mutant wounds close slower than controls. *Drp1* strong mutant wounds initially contract but start to expand after 40 mpw. At 120–130 mpw wounds start to contract again. (G) Graph of average wound area in control, *Drp1*-mild and *Drp1*-strong mutants in the first 30 mpw, corresponding to the grey region in F. Significant differences between control and *Drp1* mutants start at 4 mpw in *Drp1*-mild mutants and at 10 mpw in *Drp1*-strong mutants. A two-way ANOVA with a Tukey's correction for multiple comparisons was used to test for significant differences between groups in G. Asterisks (*) refer to control and *Drp1*-mild mutants’ comparisons. Number signs (#) refer to control and *Drp1*-strong mutant comparisons. Dashed lines depict an interval of points in which the comparison between groups gives the same degree of statistical significance, given by the symbols above. #, *P*≤0.05, **; ##, *P*≤0.01; ***P≤0.001; ****P≤0.0001. Error bars represent s.e.m. Number of embryos per condition is shown in each graph. (H,I) Maximum Z projections of the epidermis of control (H) and Drp1-overexpressing (*UAS-Drp1*) (I) embryos expressing an F-actin marker (*mCherry::Moesin*) ubiquitously under the control of the *da-Gal4* driver during wound closure. The wound-closure dynamics are similar between the two groups. (J) Graph of wound closure time in control and *UAS-Drp1* embryos. Unpaired *t*-test with Welch's correction was performed to test for significant differences between groups. (K) Graph of average wound area in control and *UAS-Drp1* embryos over time. No significant difference was found between control and Drp1-overexpressing embryos, neither in the time of wound closure nor the wound closure dynamics. A two-way ANOVA with a Sidak correction for multiple comparisons was used to test for significant differences between groups. ns, not significant (*P*>0.05). Error bars represent s.e.m. Number of embryos per condition is shown in each graph. Scale bars: 20 µm.

To understand the role of Drp1 in wound healing, we used spinning-disk microscopy to image control and *Drp1* mutant embryos expressing *GFP::Moesin* (Kiehart et al., 2000), an F-actin marker, and followed the dynamics of closure (Movie 1). Control embryos accumulate F-actin at the wound edge (Fig. 2A) and the wound area progressively decreases until the hole is closed (Fig. 2A,F). Although the initial area was similar in both conditions (Fig. 2D), *Drp1* mutant wounds took on average 128±34 min to close, significantly longer than controls (56±17 min) (Fig. 2E). In milder cases, *Drp1* mutant wounds closed at a slower rate (Fig. 2B,F). In other cases (three out of 13 *Drp1* mutant embryos), the phenotype was stronger; although the wound contracted for about 40 min post-wounding (mpw), its area began to increase again until 120–130 mpw (Fig. 2C,F). After this expansion phase, wounds contracted again, and in one case it was almost closed by the end of imaging (Fig. 2C 180 mpw, F). We quantified the wound area of control and *Drp1* mutants in the first 30 mpw and found significant differences in the first minutes after wounding (4 mpw and 10 mpw for mild and strong conditions, respectively) (Fig. 2G).

Our results suggest that inhibition of mitochondrial fission impairs wound closure. We next asked whether the induction of mitochondrial fission has the opposite effect, accelerating the wound healing process. After validating that the overexpression of Drp1 leads to induction of mitochondrial fission, resulting in a fragmented mitochondrial network (Fig. S1), we compared controls and embryos overexpressing Drp1 (*UAS-Drp1*) and expressing the F-actin marker *mCherry::Moesin* (Millard and Martin, 2008) under the control of a ubiquitous driver (*da-Gal4*) and followed the wound closure dynamics over time (Fig. 2H,I). We found no significant differences in either the time of wound closure (Fig. 2J) or in the wound area over time (Fig. 2K). This suggests that increased fission does not have an impact on wound closure dynamics.

These results show that, while Drp1 overexpression has no effect on wound closure, Drp1 loss-of-function impairs wound healing, suggesting that mitochondrial fission is necessary for wound repair regulation.

### Wounding induces no major changes in mitochondrial morphology

Our previous results suggest that mitochondrial fission is required for proper wound healing, so we wondered whether wounding triggers changes in mitochondrial morphology, towards a more fragmented mitochondrial network.

**Fig. 3.**
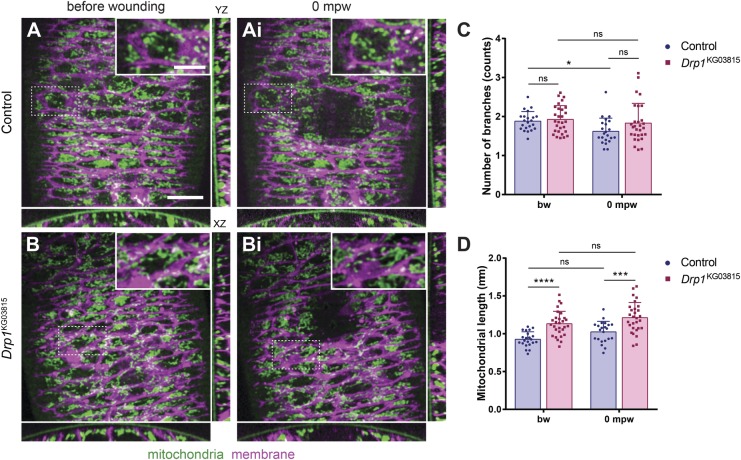
**Wounding does not induce major changes in mitochondrial morphology.** (A–Bi) Maximum Z projections of the epidermis of control (A,Ai) and *Drp1* (B,Bi) mutant embryos expressing ubiquitous mitochondrial (*EYFP::mit*o, green) and membrane (*PLCγPH::ChFP*, magenta) markers. XZ and YZ sections are shown below and on the right, respectively. Insets show a zoom of the dashed region of the respective image. Scale bar: 10 µm. Inset scale bar: 5 µm. (C) Graph of average number of branches in control and *Drp1* mutants, before and upon wounding. Control and *Drp1* mutants show similar numbers of mitochondrial branches. Wounding leads to a reduction of branching in controls but not in *Drp1* mutants. (D) Graph of average mitochondrial length in control and *Drp1* mutants, before and upon wounding. *Drp1* mutant mitochondrial network has increased length, compared to controls, both before and after wounding. Mitochondria from wounded epidermis show a similar length compared to unwounded, in both control and *Drp1* mutants. A Mann-Whitney *U* test was used to test for significant differences between groups. ns, not significant (*P*>0.05); *P=0.0275; ***P=0.0001; ****P<0.0001. *n*(control)=22 cells from nine embryos, *n*(*Drp1*)=29 cells from 12 embryos. Error bars represent s.d., bw, before wounding; mpw, minutes post wounding.

To analyse mitochondrial morphology, we used embryos expressing mitochondria (*EYFP::mito*, Lajeunesse et al., 2004) and membrane (*PLCγPH::ChFP*, Herszterg et al., 2013) markers and compared control and *Drp1* mutant embryos. *Drp1* mutants showed longer mitochondria than controls both before and upon wounding (Fig. 3A,B). Mitochondrial morphology quantification confirmed that the mitochondrial length (Fig. 3C) was higher in *Drp1* mutants than in controls, while the number of branches was similar (Fig. 3D), both before and upon wounding. In control embryos, wounding led to a reduction in the number of mitochondrial branches (Fig. 3D) but the overall mitochondrial length was unaffected (Fig. 3C), suggesting that wounding does not lead to major mitochondrial morphology changes.

Regarding localization and apicobasal distribution of mitochondria inside the cell, we did not observe major differences between control and *Drp1* mutants, either before or upon wounding (Fig. 3A–Bi, XZ and YZ sections).

Our results suggest that, although wounding does not strongly influence mitochondrial morphology, an elongated mitochondrial network such as that seen in *Drp1* mutants is detrimental for wound healing.

### *Drp1* mutants have F-actin defects during wound closure

Although cells can compensate for the loss of the actomyosin cable (Ducuing and Vincent, 2016), this structure is one of the main driving forces for wound healing (Zulueta-Coarasa and Fernandez-Gonzalez, 2017). Therefore, we checked whether the wound healing phenotype in *Drp1* mutants was associated with actomyosin cable defects. We imaged control and *Drp1* mutant embryos expressing *GFP::Moesin* (Kiehart et al., 2000) and *Zip::GFP* (Lye et al., 2014) to compare their F-actin and myosin levels.

**Fig. 4.**
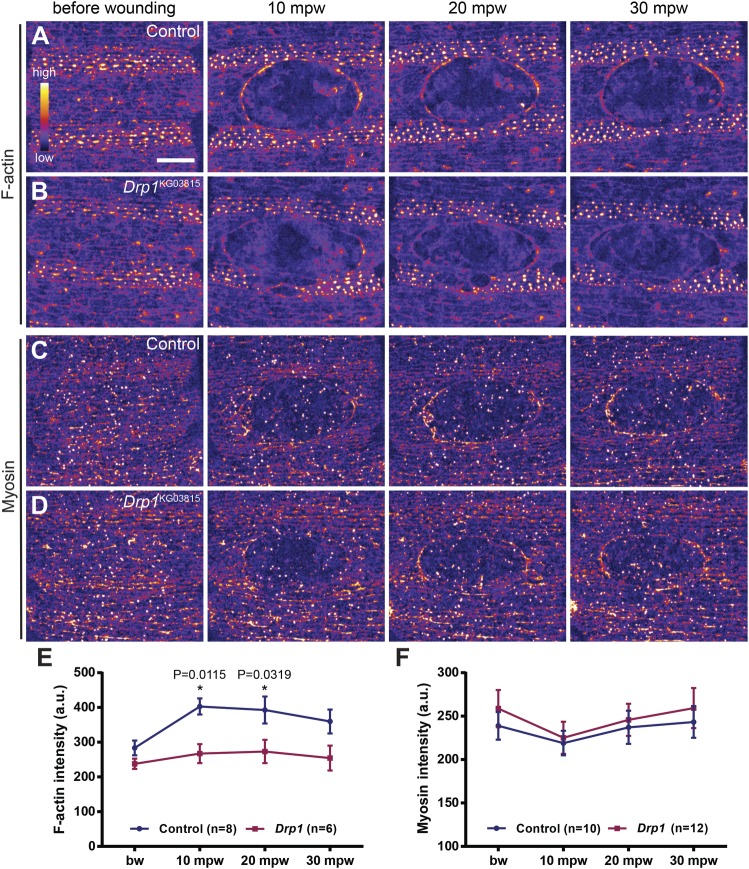
***Drp1* mutants show actin defects during wound closure.** (A–D) Maximum Z projections of the epidermis of control (A,C) and *Drp1* (B,D) mutant embryos expressing an F-actin (*GFP::Moesin*) (A,B) and a Myosin (*Zip::GFP*) (C,D) marker before and after wounding. Images are pseudo-colored with a gradient of fluorescence intensity, ranging from blue (low) to yellow (high). Although no differences between controls and *Drp1* mutants are evident before wounding, *Drp1* mutant embryos accumulate less F-actin at the wound edge than controls (compare A,B). Myosin accumulation at the wound edge seems similar between control and *Drp1* mutant embryos (compare C,D). Scale bar: 20 µm. (E) Graph of average F-actin intensity at the cell cortex before wounding and at the wound edge. F-actin levels are significantly reduced in *Drp1* mutants at 10 and 20 mpw. (F) Graph of average Myosin intensity at the cell cortex before wounding and at the wound edge. No significant differences were found between control and *Drp1* mutants. A two-way ANOVA with a Sidak correction for multiple comparisons was used to test for significant differences between groups in E and F. Only significant differences (*P*≤0.05) are represented. *P<0.05. Error bars represent s.e.m. Number of embryos per condition is shown in each graph. a.u., arbitrary units; bw, before wounding; mpw, minutes post wounding.

Both controls and *Drp1* mutant embryos accumulated F-actin (Fig. 4A,B) and myosin (Fig. 4C,D) at the wound edge. However, F-actin levels were lower in *Drp1* mutants when compared to controls (Fig. 4E). We found no significant differences in myosin levels between *Drp1* mutant and control embryos (Fig. 4F). These results suggest that the wound healing phenotype in *Drp1* mutants might be caused by defects in F-actin but not in myosin levels.

The formation of the actomyosin cable depends on remodelling of the adherens junctions (AJs) (Abreu-Blanco et al., 2012a; Carvalho et al., 2014; Hunter et al., 2015; Matsubayashi et al., 2015). After wounding, the AJ protein E-cadherin (E-cad) is downregulated at the cell boundaries facing the wound, remaining only at the lateral junctions of leading-edge cells. To test whether the F-actin defects observed in *Drp1* mutants were associated with E-cad remodelling defects, we imaged control and *Drp1* mutant embryos expressing *ubi-E-cad::GFP* (Oda and Tsukita, 1999) and *mCherry::Moesin* (Millard and Martin, 2008) before and upon wounding. We observed no significant differences in E-cad levels of control and *Drp1* mutant embryos, either before or after wounding (Fig. S2).

In summary, we propose that Drp1 regulates F-actin dynamics during wound closure, independently of AJs remodelling.

### *Drp1* mutants have altered cytosolic and mitochondrial calcium dynamics

The first signal to be detected upon wounding is an intracellular Ca^2+^ burst (Antunes et al., 2013; Razzell et al., 2013; Sammak et al., 1997; Xu and Chisholm, 2011; Clark et al., 2009). This Ca^2+^ increase regulates many wound-healing steps, including actomyosin cable formation (Antunes et al., 2013; Xu and Chisholm, 2011). Mitochondria are known regulators of Ca^2+^ homeostasis (Finkel et al., 2015; Giorgi et al., 2008; Rizzuto et al., 2012), so we asked whether the F-actin defects observed upon Drp1 loss-of-function could result from impaired Ca^2+^ dynamics.

**Fig. 5.**
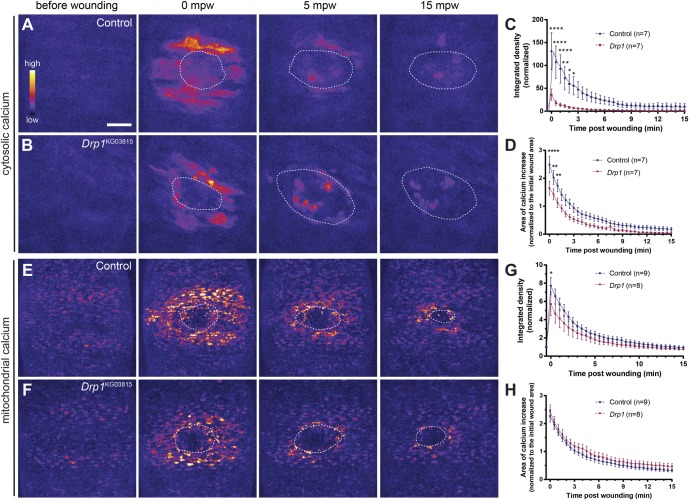
***Drp1* mutants show altered cytosolic and mitochondrial Ca^2+^ dynamics.** (A,B) Maximum Z projections of the epidermis of control (A) and *Drp1* (B) mutant embryos expressing a cytosolic Ca^2+^ sensor (*GCaMP6f*) before and after wounding. Both control and *Drp1* mutant cells around the wound dramatically increase cytosolic Ca^2+^ levels immediately upon wounding (0 mpw). Intensity returns to pre-wound levels after 15 min. Ca^2+^ levels and area of cells that respond to the wound are lower in *Drp1* mutants (B, 0 mpw) compared to controls (A, 0 mpw). (C) Graph of cytosolic Ca^2+^ intensity shows that cytosolic Ca^2+^ is lower in *Drp1* mutants compared to controls in the first 2.5 mpw. (D) Graph of average area of elevated cytosolic Ca^2+^ shows that the Ca^2+^ burst area is lower in *Drp1* mutants compared to controls from 0 to 1 mpw. (E,F) Maximum Z projections of the epidermis of control (E) and *Drp1* mutant (F) embryos expressing a mitochondrial Ca^2+^sensor (*mito::GCaMP3*) before and after wounding. Wounding triggers an increase in mitochondrial Ca^2+^ levels in both control and *Drp1* mutant cells around the wound (E,F at 0 mpw). (G) Graph of mitochondrial Ca^2+^ intensity in control and *Drp1* mutants. *Drp1* mutants have a reduced mitochondrial Ca^2+^ burst at 0 mpw, compared to controls. (H) Graph of average area of elevated mitochondrial Ca^2+^ in controls and *Drp1* mutant embryos. No significant differences were found between control and *Drp1* mutants. Images are pseudo-colored with a gradient of fluorescence intensity, ranging from blue (low) to yellow (high). Dashed lines show the wound boundaries. Scale bar: 20 µm. A two-way ANOVA with a Sidak correction for multiple comparisons was used to test for significant differences between groups in C, D, G and F. Only significant differences are represented: *P≤0.05, **P≤0.01, ****P≤0.0001. Error bars represent s.e.m. Number of embryos per condition is shown in each graph. bw, before wounding; mpw, minutes post wounding.

We imaged embryos expressing the GCaMP6f Ca^2+^ sensor (Chen et al., 2013) and measured Ca^2+^ levels before and upon wounding (Movie 2). As previously described (Razzell et al., 2013), wounding induces a dramatic and transient increase in cytosolic Ca^2+^ (cytCa^2+^) levels in the cells around the wound, that propagates in a wave-like manner reaching about two to five cell layers away from the wound, depending on the wound size (Fig. 5A). In *Drp1* mutant embryos, the cytCa^2+^ burst was less pronounced than in controls (Fig. 5B,C). Moreover, the area in which Ca^2+^ increase was observed was significantly reduced in *Drp1* mutants compared to controls (Fig. 5D), suggesting that impairing Drp1 function affects not only Ca^2+^ levels but also the intercellular Ca^2+^ propagation.

Mitochondria can uptake Ca^2+^ from the cytosol, thereby modulating cytCa^2+^ (Szabadkai and Duchen, 2008). As mitochondrial morphology influences mitochondrial Ca^2+^ (mitCa^2+^) levels (Bianchi et al., 2006; Gerencser and Adam-Vizi, 2005; Szabadkai et al., 2004), we examined control and *Drp1* mutant embryos expressing a mitochondria-targeted GCaMP3 Ca^2+^ sensor (*mito::GCaMP3*, Lutas et al., 2012) before and upon wounding (Movie 3). Similar to what was detected for cytCa^2+^, we observed an increase in mitCa^2+^ around the wound in both control (Fig. 5E) and *Drp1* mutant (Fig. 5F) epidermis. Quantification of mitCa^2+^ intensity showed a reduced response upon wounding in *Drp1* mutant embryos compared to controls (Fig. 5G, 0 mpw). This reduction is not as dramatic as that seen for cytCa^2+^, which may be due to the different sensitivity of the cytCa^2+^ sensor. No differences were found in the area of increased mitCa^2+^ (Fig. 5H), suggesting that only the cytCa^2+^ propagation is affected.

To further investigate the impact of mitochondrial fission impairment in wound closure, we tested how another component of the fission machinery regulates wound healing. We knocked down Fis1, a Drp1 receptor (Losón et al., 2013), by ubiquitously expressing RNAi against Fis1 and analysing its effect on Ca^2+^ and F-actin dynamics upon wounding (Fig. S3). Both the cytCa^2+^ and mitCa^2+^ bursts upon wounding were reduced in Fis1 RNAi-expressing embryos compared to controls (Fig. S3A,B,C,E,F,G), although no effect on Ca^2+^ propagation across the epidermis was detected (Fig. S3D,H). On the other hand, no significant differences in F-actin levels were found between Fis1 knockdown and control embryos (Fig. S3I–K). These results support the hypothesis that mitochondrial fission regulates Ca^2+^ dynamics during wound closure.

### *Drp1* mutants show reduced mitochondrial ROS production upon wounding

Ca^2+^-dependent ROS production upon wounding has been shown to regulate the F-actin cytoskeleton and the wound healing response both in *Caenorhabditis elegans* and *Drosophila* (Hunter et al., 2018; Xu and Chisholm, 2014). Mitochondria are known sources of ROS (Murphy, 2009) and their ability to produce them can be regulated by mitochondrial dynamics. Fragmentation of the mitochondrial network is associated with increased ROS production and inhibition of mitochondrial fission can reduce oxidative stress (Galloway et al., 2012). As our results show that Drp1 regulates Ca^2+^ and F-actin dynamics during wound healing, we hypothesized that ROS production might be the link between these two wound closure events. To investigate whether *Drp1* mutants have altered ROS production, we imaged control and *Drp1* mutant embryos expressing a genetically-encoded ratiometric mitochondrial green fluorescent protein that shifts irreversibly to red fluorescence when oxidized (Laker et al., 2014).

**Fig. 6.**
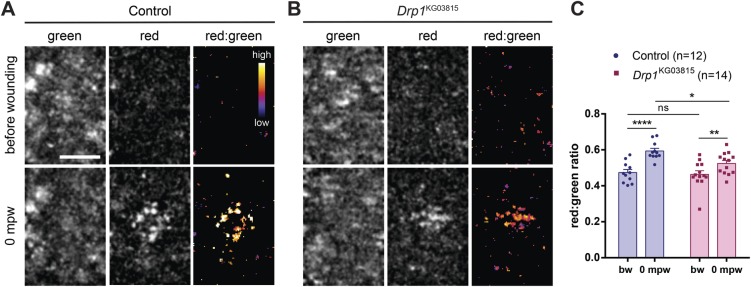
***Drp1* mutant embryos show reduced mitochondrial ROS production upon wounding.** (A,B) Maximum Z projections of the wound region of control (A) and *Drp1*-mutant (B) embryos expressing the mitochondrial ROS sensor *MitoTimer* ubiquitously, before and upon wounding (0 mpw). This reporter gene encodes a protein that irreversibly changes its fluorescence spectrum from green to red upon oxidation. Images show the green and red channels for each embryo, as well as the red:green ratio after image processing. Red:green ratio images are pseudo-colored with a gradient of fluorescence intensity, ranging from blue (low) to yellow (high). Scale bar: 10 µm. (C) Graph of the average red:green ratio of control and *Drp1* mutant embryos, before and after wounding (0 mpw). Red:green ratio is a measure of ROS levels. Pre-wound ROS levels are similar between control and *Drp1* mutants. Wounding increases ROS levels, both in controls and *Drp1* mutants but this increase is lower in *Drp1* mutant embryos compared to controls. A two-way ANOVA with a Sidak correction for multiple comparisons was used to test for significant differences between groups. ns, not significant, *P=0.0170, **P=0.0049, ****P<0.0001. Error bars represent s.e.m. Number of embryos per condition is shown in the graph.

Upon wounding, we observed an increase in the red signal, an indicator of ROS production, both in control and in *Drp1* mutants (Fig. 6A,B). To quantify ROS production, we calculated the red:green fluorescence intensity ratio and observed that the ROS levels produced upon wounding were significantly lower in *Drp1* mutants compared to control embryos (Fig. 6C).

Our results show that impairing mitochondrial fission leads to reduced mitochondrial ROS production in response to wounding.

### The Rho GTPase effector Pkn is downregulated at the wound edge in *Drp1* mutants

To further investigate the mechanisms through which mitochondrial fission regulates the wound-healing response, we assessed whether known F-actin modulators were dysregulated in *Drp1* mutants. F-actin dynamics and myosin contractility at the wound edge have been shown to be modulated by members of the Rho family of GTPases, Rho1, Rac and Cdc42 (Verboon and Parkhurst, 2015). Moreover, the regulation of these GTPases is mediated by Ca^2+^ and ROS triggered upon wounding (Soto et al., 2013; Xu and Chisholm, 2014). Rho and its effectors coordinate actomyosin cable formation and contractility, while Rac and Cdc42 are more important to form F-actin protrusions that coordinate cell migration at the leading edge and the final knitting of the epithelium at the end of closure (Abreu-Blanco et al., 2012b; Verboon and Parkhurst, 2015).

**Fig. 7.**
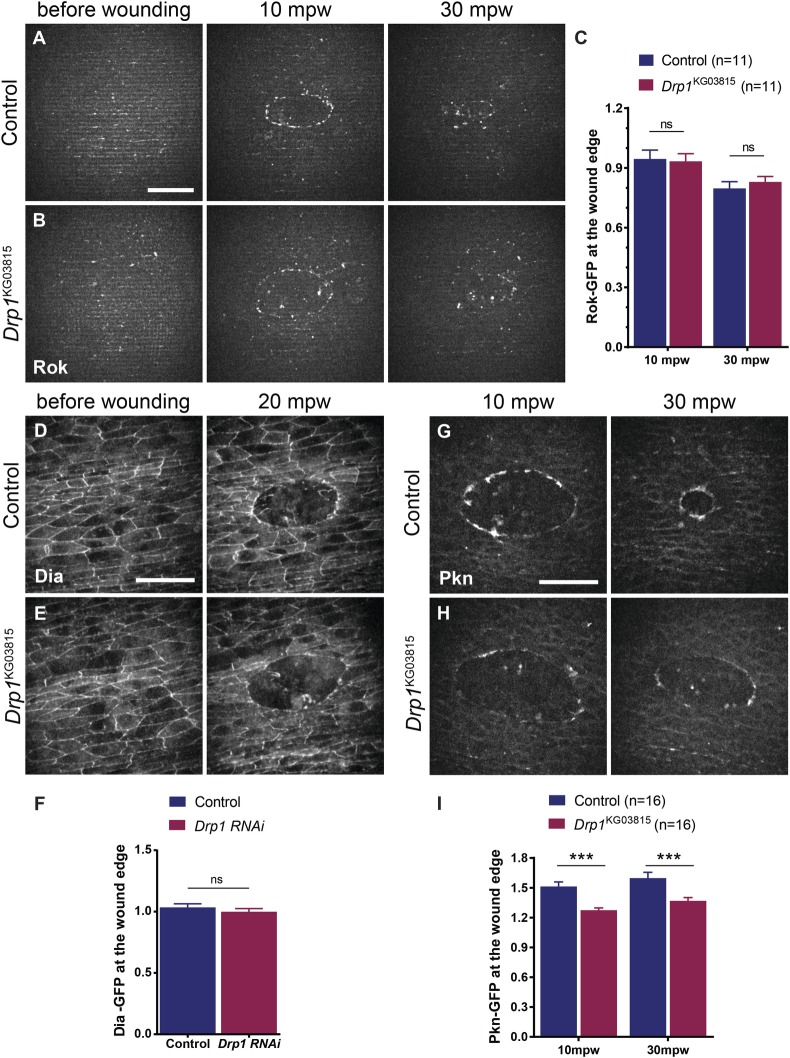
***Drp1* mutants show reduced accumulation of Pkn at the wound edge, but not of Rok** **or** **Dia.** (A,B) Maximum Z projections of control (A) and *Drp1*-mutant (B) embryos expressing the *Rok::GFP*, before wounding and at 10 and 30 mpw. (C) Graph of the average *Rok::GFP* intensity at the wound edge in controls and *Drp1* mutants at 10 and 30 mpw, normalized to pre-wound levels. No significant differences in Rok accumulation at the wound edge were found between controls and *Drp1* mutants. (D,E) Maximum Z projections of control (D) and *Drp1* mutants (E) expressing the *Dia::GFP*, before wounding and at 20 mpw. (F) Graph of the average *Dia::GFP* intensity at individual cell junctions at the wound edge in control and *Drp1*-mutant embryos at 20 mpw, normalized to their respective intensity before wounding. No significant differences in Dia localization at the wound edge were found between controls and *Drp1* mutants. (G,H) Maximum Z projections of control (G) and *Drp1*-mutant (H) embryos expressing the *Pkn-GFP* at 10 and 30 mpw. (I) Graph of the average *Pkn-GFP* intensity at the wound edge in controls and *Drp1* mutants at 10 and 30 mpw, normalized to background levels. *Drp1* mutants show a reduced accumulation of Pkn at the wound edge compared to controls. Error bars represent s.e.m. A two-way ANOVA with a Sidak correction for multiple comparisons was used to test for significant differences between groups in C and I. A Wilcoxon test was used to test for significant differences between groups in F. The number of embryos per condition is shown in C and I. In F, *n*(Control)=82 junctions from nine embryos and *n*(Drp1)=99 cell junctions from ten embryos. Scale bars: 20 µm. mpw, minutes post wounding.

As we observed defects in the F-actin accumulation at the wound edge in *Drp1* mutants, we decided to focus on Rho effectors: Rho kinase (Rok), Diaphanous (Dia) and Protein kinase N (Pkn). Rok activates the myosin regulatory light chain, directly by phosphorylation or by inactivation of myosin phosphatases, thus promoting actomyosin contractility (Kimura et al., 1996; Ueda et al., 2002). Dia is a formin that promotes the polymerization of unbranched F-actin (Narumiya et al., 1997). Pkn has been implicated in the regulation of cell migration, but how it exerts its function is not fully understood (Lachmann et al., 2011; Lim et al., 2004). We imaged embryos expressing GFP-tagged versions of the Rok, Dia and Pkn (Fig. 7).

For the analysis of Rok and Pkn, we compared controls with *Drp1* mutant embryos, while for Dia we used RNAi to knockdown Drp1. Out of the three Rho1 effectors, only Pkn showed a significant reduction in its accumulation at the wound edge in *Drp1* mutants when compared to controls (Fig. 7D–F). The accumulation of Rho1 effectors at the wound edge is a consequence of their activation by Rho1, so our results suggest that Drp1 loss-of-function leads to a reduction in Rho1 activity upon wounding.

In summary, we have identified mitochondrial-dynamics proteins as novel embryonic wound-healing regulators. Our data show that inhibition of mitochondrial fission by Drp1 loss-of-function leads to defects in Ca^2+^, ROS and F-actin dynamics upon wounding, culminating in wound-healing impairment.

## DISCUSSION

Epithelial tissues are critical to protect us from the external environment (Lowe and Anderson, 2015). Understanding how epithelial tissues drive efficient wound repair is of the upmost importance for the biomedical field. In this study, we used a model of simple epithelial wound closure, the embryonic epidermis of the fruit fly, to uncover the role of mitochondrial dynamics in the wound-healing response. Little is known about how mitochondria contribute to tissue repair, besides their involvement in the production of ROS, which in turn can regulate the wound-healing process (Hunter et al., 2018; Sanchez et al., 2018; Xu and Chisholm, 2014). Some data support the conclusion that mitochondrial dynamics can regulate cell migration, an essential process in wound repair (Ko et al., 2017; Zhao et al., 2013), but whether this is relevant for embryonic wound repair had never been addressed.

We started by performing a genetic screen to understand whether the mitochondrial dynamics machinery is required for wound closure. Mitochondrial dynamics is mediated by large GTPases, namely Drp1, that mediates mitochondrial fission; and Opa1 and Mfns (Marf in *Drosophila*) that control the fusion of the mitochondrial membranes. Out of the tested four *Marf* alleles and miRNA-mediated knockdown, only *Marf*^J^ mutants showed an increased number of open wounds compared to controls. Most of the *Marf* alleles have not been characterized, so it is unclear why only *Marf*^J^ mutants showed a wound-healing phenotype, being possible that this is an unspecific effect of this allele. It is also conceivable that the perdurance of maternal contribution masks the effects of Marf loss-of-function in the case of the remaining *Marf* alleles. On the other hand, both *Opa1* mutant alleles showed a significant wound-healing phenotype, favouring the hypothesis that mitochondrial fusion is necessary for proper embryonic wound healing. Future studies focusing on mitochondrial fusion are important to complement our results. Regarding mitochondrial fission, *Drp1*^KG03815^, a loss of function allele, showed the strongest phenotype compared with the other *Drp1* mutant alleles. Not much detail is available about how the different mutations affect Drp1 function. However, all different heteroallelic combinations resulted in an increase in open wounds, which indicates that the Drp1 loss-of-function phenotype is consistent. Other players of mitochondrial fission, Fis1 and Gdap1, are also required for wound healing, as their mutation leads to an increased number of unhealed wounds. Altogether, these data strongly implicate mitochondrial fission in the regulation of epithelial repair.

We found that the wound-closure dynamics of *Drp1* mutants is significantly affected, as these embryos take more than two times longer to close the wounds than control embryos. We observed two degrees of wound closure phenotypes: a mild phenotype, characterized by a slower wound closure rate than in controls; and a strong phenotype, in which the wound area expands. It is unclear why some wounds present this expansion phase, but a similar phenotype has been described in mutants for a component of the invertebrate occluding junctions (OJs). OJ disruption leads to defects in the actomyosin cable, cellular shapes and rearrangements as well as in tissue mechanical properties (Carvalho et al., 2018). It would be interesting to explore the link between mitochondrial dynamics and OJs in wound healing in future studies.

A recent study in the *C. elegans* epidermis showed that increased mitochondrial fission, either by impairing fusion or by drug-mediated fission induction, led to accelerated wound closure (Fu et al., 2020). Interestingly, we observed no difference in wound-healing dynamics between Drp1-overexpressing embryos and controls. Moreover, we observed no major differences in mitochondrial morphology between wounded and unwounded embryos, while in *C. elegans* wounding triggers mitochondrial fission (Fu et al., 2020). It seems that mitochondrial dynamics can have different effects on wound healing, and vice versa, depending on the context. In the case of the *Drosophila* embryonic epidermis, an elongated mitochondrial network, such as observed in *Drp1* mutants, is prejudicial for wound healing, while increasing the fragmentation of mitochondria has no impact on the wound-closure rate. We thus propose that there is an optimal mitochondrial morphology that favours proper wound closure.

To understand the mechanism through which mitochondrial fission regulates wound healing, we characterized the known wound-healing events in *Drp1* mutants, such as the formation of the actomyosin cable at the wound edge (Kiehart et al., 2000; Wood et al., 2002; Xu and Chisholm, 2011). *Drp1* mutants show defects in the F-actin accumulation at the wound edge, which may be the cause of the observed wound-healing impairment.

The formation of the actomyosin cable is known to depend on several factors, such as an intracellular Ca^2+^ increase upon wounding (Antunes et al., 2013; Razzell et al., 2013; Xu and Chisholm, 2011), the remodelling of the AJs (Abreu-Blanco et al., 2012a; Carvalho et al., 2014; Hunter et al., 2015), ROS production (Hunter et al., 2018; Xu and Chisholm, 2014) and the activation of the Rho family of GTPases and their targets (Brock, 1996; Verboon and Parkhurst, 2015; Wood et al., 2002).

In addition to the established cytCa^2+^ burst, we also observed a rapid increase in mitCa^2+^ levels upon wounding, consistent with what has been seen in *C. elegans* wound repair (Xu and Chisholm, 2014), suggesting that, similarly to the cytCa^2+^ burst, this is a conserved response to tissue injury. Remarkably, both wound-induced cytCa^2+^ and mitCa^2+^ bursts were reduced upon Drp1 loss-of-function, suggesting that mitochondrial fission strongly impacts on Ca^2+^ dynamics during wound closure. It is well established that mitochondria can take up Ca^2+^ from the cytosol, thereby modulating cytCa^2+^levels (Szabadkai and Duchen, 2008). However, the relationship between cytCa^2+^ and mitCa^2+^ in the context of wound healing is not clear. Injury triggers Ca^2+^ influx from the extracellular environment (Antunes et al., 2013; Razzell et al., 2013; Xu and Chisholm, 2011). The elevated cytCa^2+^ levels induce Ca^2+^ release from the endoplasmic reticulum (ER) mediated by the inositol-3-phosphate (IP3) receptor (IP3R), followed by propagation of Ca^2+^ and IP3 to neighbouring cells through gap junctions (Narciso et al., 2015; Razzell et al., 2013; Restrepo and Basler, 2016). In other cellular contexts, mitochondria localize close to the ER, forming Ca^2+^ signalling microdomains. Ca^2+^ uptake by mitochondria reduces the cytCa^2+^ levels close to the open ER channels (local cytCa^2+^), preventing their Ca^2+^-dependent inactivation. By controlling ER Ca^2+^ channels activity, mitCa^2+^ uptake affects global cytCa^2+^ (Billups and Forsythe, 2002; Rizzuto et al., 2012). Our results lead us to speculate that in *Drp1* mutants the Ca^2+^ buffering capacity of mitochondria is compromised, leading to IP3R inhibition and lower global cytCa^2+^ levels. This could also affect the Ca^2+^ wave propagation, as less Ca^2+^ and/or IP3 would cross gap junctions. Indeed, *Drp1* mutants showed a reduction in the area of Ca^2+^ increase, which may indicate that the Ca^2+^ wave propagation is affected.

Mitochondrial shape, number and distribution can affect their contacts with the ER and impact on mitCa^2+^ propagation. Based on previous studies, the inhibition of Drp1 with consequent elongated mitochondrial network should favour the proximity with the ER and facilitate mitCa^2+^ uptake (Cieri et al., 2018; Szabadkai et al., 2006). Here, we observe the opposite effect, suggesting that the role of Drp1 on mitCa^2+^ regulation may be context dependent. Further work is needed to understand how Drp1 regulates mitochondrial Ca^2+^ uptake in the *Drosophila* epidermis.

The wound-induced Ca^2+^ burst triggers the production of mitochondrial ROS, which then regulates the actomyosin cable formation, either by activating RHO-1 or by regulating AJ remodelling (Hunter et al., 2018; Xu and Chisholm, 2014). Our results show that the absence of functional Drp1 leads to reduced ROS production. No difference in E-cad remodelling at the wound edge was observed in *Drp1* mutants, so we hypothesized that Rho1 activation could be affected in our model. As a readout of Rho1 activation we assessed the localization of three of its effectors: Rok, Dia and Pkn. Rok accumulation at the wound edge was unaffected in *Drp1* mutants compared to controls. As Rok regulates myosin activation and contractility, this result could explain why myosin levels were similar between controls and *Drp1* mutants. Dia has been shown to regulate F-actin dynamics in simple epithelia wound healing (Antunes et al., 2013; Matsubayashi et al., 2015), but we observed no differences in Dia localization between control and *Drp1* mutant embryos. Surprisingly, we found that Drp1 loss-of-function leads to a significant reduction in Pkn accumulation at the wound edge. Pkn has been implicated in the regulation of cellular shape changes associated with dorsal closure, a developmental process that shares similarities with wound healing (Lu and Settleman, 1999), but its function in the context of wound repair has never been addressed. *In vitro* studies have shown that Pkn interacts with the F-actin regulator alpha-actinin, in a Ca^2+^-dependent manner (Mukai et al., 1997), and that it can regulate F-actin during cell migration (Lim et al., 2004). Thus, our results point to a novel player in regulating F-actin dynamics downstream of Rho1 and mitochondrial dynamics during wound closure.

As it has been shown that Ca^2+^ regulates F-actin dynamics upon wounding (Antunes et al., 2013; Xu and Chisholm, 2011), the Ca^2+^ defects observed in *Drp1* mutants could be the cause of the reduced ROS production, defective F-actin accumulation at the wound edge and consequent wound healing impairment. Nevertheless, we cannot exclude the possibility that Drp1 regulates F-actin in a Ca^2+^-independent manner. The knockdown of Fis1, a Drp1 receptor, led to defects in Ca^2+^ dynamics, similar to the Drp1 loss-of-function phenotype, but not in F-actin dynamics. This could be due to redundancy of Drp1 receptors (Losón et al., 2013), to an incomplete knockdown of Fis1, to off-site mutations in the *Drp1*-mutant allele or by a direct, Ca^2+^-independent, link between Drp1 and F-actin. Drp1 can directly bind F-actin (DuBoff et al., 2012; Ji et al., 2015), but whether Drp1 can directly regulate F-actin dynamics is not known. Drp1 knockdown also reduces the formation of actin protrusions and invasiveness of glioma cells by regulating the RHOA/ROCK pathway (Yin et al., 2016), known to regulate cytoskeletal dynamics (Amano et al., 2010). Future studies should investigate how Drp1 controls F-actin dynamics during wound closure.

Other mitochondrial functions, such as metabolism, may have an impact on wound healing. A previous screen identified two genes required for embryonic wound healing that are related to mitochondrial metabolism, but their function remains unexplored (Campos et al., 2010). Moreover, changes in mitochondrial morphology are linked to metabolism (Wai and Langer, 2016), so the relationship between these processes in the context of wound healing is worth investigating.

In conclusion, our work shows a novel role for mitochondrial dynamics in epithelial repair. In particular, mitochondrial fission is essential for wound-induced Ca^2+^ and ROS increase and F-actin polymerization at the wound edge during epithelial repair.

## MATERIALS AND METHODS

### *Drosophila* strains and genetics

Flies were maintained at 25°C on standard *Drosophila* medium, except for experiments using RNAi, which were performed at 29°C. The fly lines used in the wounding assay were: *UAS-Marf miRNA* (67158), *Marf*^B^ (67154), *Marf*^E^ (67155), *Marf*^I^ (57097), *Marf*^J^ (57096), *Opa1*^s3475^ (12188), *Opa1*^EY09863^ (20054), *Fis1*^MI10520^ (55496), *Gdap1*^MB07860^ (25575), *Drp1*^KG03815^ (13510), *Drp1^D20^* (3911), *Drp1*^T26^ (3662), *Df(2L)ED134* (8900) and *Df(2R)Exel7131* (7876). *w*^1118^ flies were used as controls for the wounding assay.

The live reporter lines used were: *sqh-EYFP::mito* (7194) (Lajeunesse et al., 2004) and *UAS-mito::GFP* (8442) (Rizzuto et al., 1995), which express a mitochondrial targeting signal to mark mitochondria tagged with EYFP/GFP; *ubi-PLCγPH::ChFP* (Herszterg et al., 2013), which expresses the PLCγPH domain tagged with mCherry, to mark the cell membrane; *sqh-GFP::Moesin* (59023) (Kiehart et al., 2000) or *UAS-mCherry::Moesin* (Millard and Martin, 2008), which consist of the actin-binding domain of Moesin tagged with GFP or mCherry, respectively, to mark F-actin; *Zip^CPTI-100036^-GFP* (115383) (Lye et al., 2014), a GFP-expressing protein trap, to mark myosin II; *ubi-E-cad::GFP* (109007) (Oda and Tsukita, 1999) encoding full length E-cad tagged with GFP to mark E-cad; *UAS-GCaMP6f* (52869) (Chen et al., 2013), a GFP Ca^2+^ sensor, to mark cytosolic Ca^2+^; *UAS-mito::GCaMP3* (Lutas et al., 2012), the GCaMP3 Ca^2+^ sensor fused to a mitochondrial targeting sequence, to mark mitochondrial Ca^2+^; *UAS-MitoTimer* (57323) (Laker et al., 2014), expressing a mitochondria-targeted DsRed variant that shifts from green to red when oxidized, to mark mitochondrial ROS; *sqh-GFP::Rok* (52289) (Abreu-Blanco et al., 2014), which expresses GFP-tagged Rok constitutively under control of *sqh* regulatory sequences to mark Rok; *Pkn[CC01654]-GFP* (51566) (Buszczak et al., 2007), a GFP-expressing protein trap, to mark Pkn; and *UAS-dia::EGFP* (56751), which expresses GFP-tagged Dia, to mark Dia.

*UAS-Luc* (35788) flies, which express firefly Luciferase under UAS control in the VALIUM10 vector, were used as controls for the RNAi and *UAS-Drp1* experiments. *UAS-Drp1-TRiP.HMC03230 RNAi* (51483) was used to knockdown Drp1 in Fig. 7. UAS-Fis1-TRiP.HMS05301 RNAi (63027) was used to knockdown Fis1 in Fig. S3. *UAS-Drp1* (51647) was used to overexpress Drp1 in Fig. 2. UAS lines were expressed under the control of either the e22c-Gal4 driver (1973, to label the epidermis) or the ubiquitous da-Gal4 driver (55851) (see figure legends).

*ubi-PLCγPH::ChFP* and *UAS-mito::GCaMP3* were a gift from Y. Bellaïche and F. Kawasaki, respectively. *Zip^CPTI-100036^-GFP* and *ubi-E-cad::GFP* were obtained from the Kyoto *Drosophila* Genomics and Genetic Resources Stock Center, Kyoto Institute of Technology, Kyoto, Japan. All the remaining fly lines were obtained from the Bloomington *Drosophila* Stock Center, Indiana University, Bloomington, USA. Stock centre numbers are indicated above for each line. Information on the nature of the mutant alleles and transgenes can be found on Flybase (Thurmond et al., 2019).

For live imaging, the *Drp1*^KG03815^ allele was combined with live reporter lines. Mutant alleles and recombinant lines were crossed to balancer stocks that express GFP driven by a *Twist-Gal4* driver (Halfon et al., 2002). Homozygous mutant embryos were identified by the absence of GFP fluorescence. Stage 15–16 embryos were selected by the shape of the yolk (Campos-Ortega and Hartenstein, 1985).

### Wounding assay

The wounding assay was performed as previously described (Campos et al., 2010). Fly lines were in-crossed in laying pots and embryos were collected at 25°C overnight in apple juice agar plates. Embryos were dechorionated in 50% bleach and rinsed extensively with water. Selected mutant and control embryos were mounted on double-sided tape affixed to a slide, covered with halocarbon oil 700 (Sigma-Aldrich) and a 32×32 mm coverslip, and sealed with nail polish. A 24×24 mm coverslip bridge was used between the slide and the top coverslip to avoid embryo squashing.

The embryos were wounded at 25°C by using a nitrogen laser-pumped dye laser (435 nm; Micropoint Photonic Instruments) connected to a Nikon/Andor Revolution XD spinning-disk confocal microscope with an electron-multiplying charge-coupled device (EMCCD) camera (iXon 897) using iQ software (Andor Technology) and using a 60× Plan Apochromat VC Perfect Focus System (PFS) 1.4 NA oil-immersion objective.

After wounding, the top coverslip was carefully removed and the embryos were left to recover in a humid chamber at 20°C. About 16 h later, the wounded embryos were scored under a stereo microscope for closed/intermediate/open wounds.

The percentage of open wounds was calculated as the ratio of nearly hatching embryos with open wounds over the total number of wounded embryos (dead animals and intermediate wound phenotypes were excluded).

Images of representative embryos depicting open, intermediate and closed wounds were acquired using a Zeiss Axio Imager Z2 widefield system equipped with an Axiocam 506 monochromatic CCD camera, a 10× EC Plan-Neofluar 0.3 NA objective and the Zen Pro 2012 software. Individual z slices with a step size of 10 µm were acquired. Stacks were processed using the Extended Depth of Field plugin based on the complex wavelet method on Fiji (Forster et al., 2004; Schindelin et al., 2012).

### Live imaging

Live imaging was performed as described previously (Carvalho et al., 2018). Dechorionated stage 15 embryos were mounted on their ventral side on glass-bottomed culture dishes (MatTek) with embryo glue (double-sided tape diluted in heptane) and covered with halocarbon oil 27 (Sigma-Aldrich). Embryos were wounded as described above for the wounding assay except that the laser power was lower in order to inflict smaller wounds that are able to close during the imaging procedure.

Time-lapse microscopy of transgenic embryos was performed at 25°C on a Nikon/Andor Revolution XD spinning-disk confocal microscope with a 512 EMCCD camera (iXon 897) with a 60× Plan Apochromat VC PFS 1.4 NA oil-immersion objective or a 60× Plan Apochromat VC PFS 1.2 NA water-immersion objective (Nikon) and using the iQ software.

Individual z slices with a step size of 0.28 µm, (Figs 2, 3 and 4; Figs S1 and S3I–J), 0.36 µm (Fig. 7, Fig. S2) or 0.5 µm (Figs 5 and 6; Fig. S3A,B,E,F), were acquired for a single time point or every 30 s, 2 min, 2.5 min or 10 min for 30–160 min. For F-actin, myosin, E-cad and Rho effectors imaging, Z stacks were acquired with frame averaging of 2. For mitochondrial morphology quantification, images were acquired with frame averaging of 4.

### Image analysis and quantifications

All images were processed and analyzed using Fiji [ImageJ, National Institutes of Health (NIH); Schindelin et al., 2012], unless stated otherwise. Z stacks were processed to obtain maximum Z projections or XZ/YZ orthogonal sections.

#### Mitochondrial morphology

*EYFP::mito* or *mito::GFP* Z stacks were deconvolved with Huygens Remote Manager (Scientific Volume Imaging, The Netherlands, http://svi.nl), using the Classic Maximum Likelihood Estimation (CMLE) algorithm, with Signal to Noise Ratio (SNR):15 and 30 iterations. Individual cells were manually outlined and cropped from maximum Z projections of deconvolved *sqh-mito-YFP* merged with *PLCγPH::ChFP* or *mCherry:Moesin* Z stacks. Wound leading-edge cells were selected and their mitochondrial morphology was compared before and immediately after wounding. Mitochondrial morphology from the selected cells was quantified using MiNA (Mitochondrial Network Analysis) 2.0.0 macro for ImageJ (Valente et al., 2017) (https://github.com/StuartLab/MiNA), selecting a Maximum Entropy Threshold method and Ridge Detection. The branch–length mean and network–branches mean output parameters for each cell were plotted. The branch–length mean, which was called mitochondrial length for simplicity, is the mean length of all the lines used to represent the mitochondrial structures. The network–branches mean is the mean number of attached lines used to represent each structure.

#### Wound area

*GFP::Moesin* Maximum Z projections were used. An ellipse was drawn along the wound edge over time, and the area was obtained using the Measure tool. For each embryo, the area was normalized relative to the initial wound area. For statistical comparisons, only the first 30 min after wounding were considered, as shortly after that wounds start to close in control embryos.

#### Fluorescence intensity measurements

To measure mitochondrial and intracellular Ca^2+^ dynamics, *GCaMP-6f* maximum projections were used after using a median filter (0.5 pixel). The wound area, measured from *mCherry::Moesin* maximum projections from respective embryos, was deleted from GCaMP-6f maximum projections to exclude the signal coming from cellular debris and wound-recruited hemocytes. The region of Ca^2+^ increase upon wounding was selected by applying an Intensity Threshold (Otsu). The Mean Grey Value, Area and Integrated Density (the product of Area and Mean Grey Value) were obtained using the Measure Tool, before and during wound closure. We plotted the Integrated density normalized to pre-wound values and the area of Ca^2+^ increase normalized to the initial wound area.

To measure F-actin, myosin and Pkn intensities at the wound edge, maximum Z projections of *mCherry::Moesin*, *Zip::GFP* and *Pkn-GFP* stacks were used after Rolling Ball Background Subtraction (15 pixel for F-actin and myosin, 10 pixel for Pkn). For F-actin and myosin, the wound edge and the cortical region of epithelial cells (ten cells per embryo) before wounding were outlined using a 3-pixel-wide segmented line, and the mean grey value was obtained using the Measure tool. For F-actin quantifications, cells containing actin-rich denticle precursor structures were excluded as they mask the actin present at the cable and cell cortex. For Pkn, the wound edge was outlined using a 5-pixel-wide segmented line and the mean grey value was obtained using the Measure tool. This value was normalized for each embryo by dividing it by the average intensity value of a 50-pixel diameter circle at the wound hole in the same embryo. The *mCherry::Moesin* channel was used to confirm the location of the wound edge.

To measure E-cad and Dia intensities, maximum Z projections of *ubi-E-cad::GFP* and *UAS-Dia::GFP* stacks, respectively, were used after Rolling Ball Background Subtraction (10 pixel for E-cad, 50 pixel for Dia). The *mCherry::Moesin* channel was used to confirm the location of the wound edge. Junctions were outlined using a 4 (for E-cad) or 5 (for Dia)-pixel-wide segmented line and the average intensity obtained using the Measure tool. To calculate the intensity decrease (fold change) at the wound edge, the intensity value for each wound edge junction after wounding (10 and 30 mpw for E-cad, 20 mpw for Dia) was divided by the intensity value obtained for the same junction before wounding.

To measure ROS production, confocal Z stacks were obtained for both green (excitation/emission 488/518 nm) and red (excitation/emission 543/572 nm) channels. A 19.92×29.04 µm region around the wound was selected and background was subtracted (rolling ball radius of 15 pixels) in both channels. The red channel positive pixels were selected by applying an Intensity Threshold (Otsu). The maximum Z projections of the red and green channels were then divided to obtain the red to green ratio and the mean fluorescence intensity was measured at two time points: before wounding and upon wounding (0 mpw).

#### Statistics

Statistical analysis was performed using GraphPad Prism 6.01 (GraphPad Software, La Jolla California, USA). Statistical tests, *P*-values, sample sizes, and error bars are indicated in the respective figure legends.

## Supplementary Material

Supplementary information
