# Supplementary material for: Drp1-mediated mitochondrial fission regulates calcium and F-actin dynamics during wound healing
Source: Biol Open. 2020 May 4;9(5):bio048629. doi: 10.1242/bio.048629 (PMC7225088; doi:10.1242/bio.048629)
Supplement: Supplementary information [file biolopen-9-048629-s1.pdf]

Figure S1

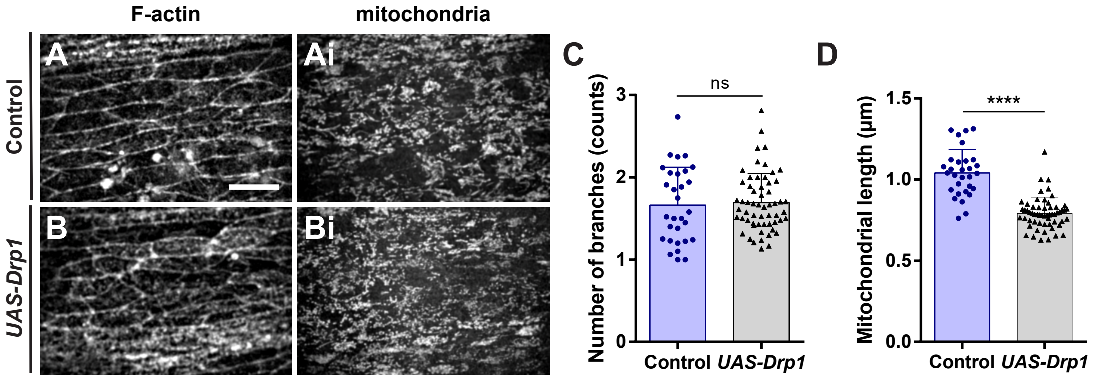

Figure S1. Drp1 overexpression results in reduced mitochondrial length. (A-Ai, B-Bi) Maximum Z projections of the epidermis of control (A-Ai) and UAS-*Drp1* (B-Bi) embryos expressing a mitochondrial (*mito::GFP*, Ai, Bi) and an F-actin (m*Cherry::Moesin,* A, B) marker ubiquitously under the control of the *da-Gal4* driver. Scale bar = 10 µm. **(C)** Graph of average number of branches in control and *UAS-Drp1* embryos. **(D)** Graph of average mitochondrial length in control and *UAS-Drp1* embryos. Unwounded control and *UAS-Drp1* embryos show similar number of mitochondrial branches but the mitochondrial length is reduced in *UAS-Drp1* embryos compared to controls. A Mann-Whitney test was used to test for significant differences between groups. ns – not significant (P > 0.05), **** P < 0.0001. N(control) = 30 cells from 6 embryos, N(UAS-*Drp1*) = 58 cells from 8 embryos. Error bars represent s.d.

Biology Open • Supplementary information

Figure S2

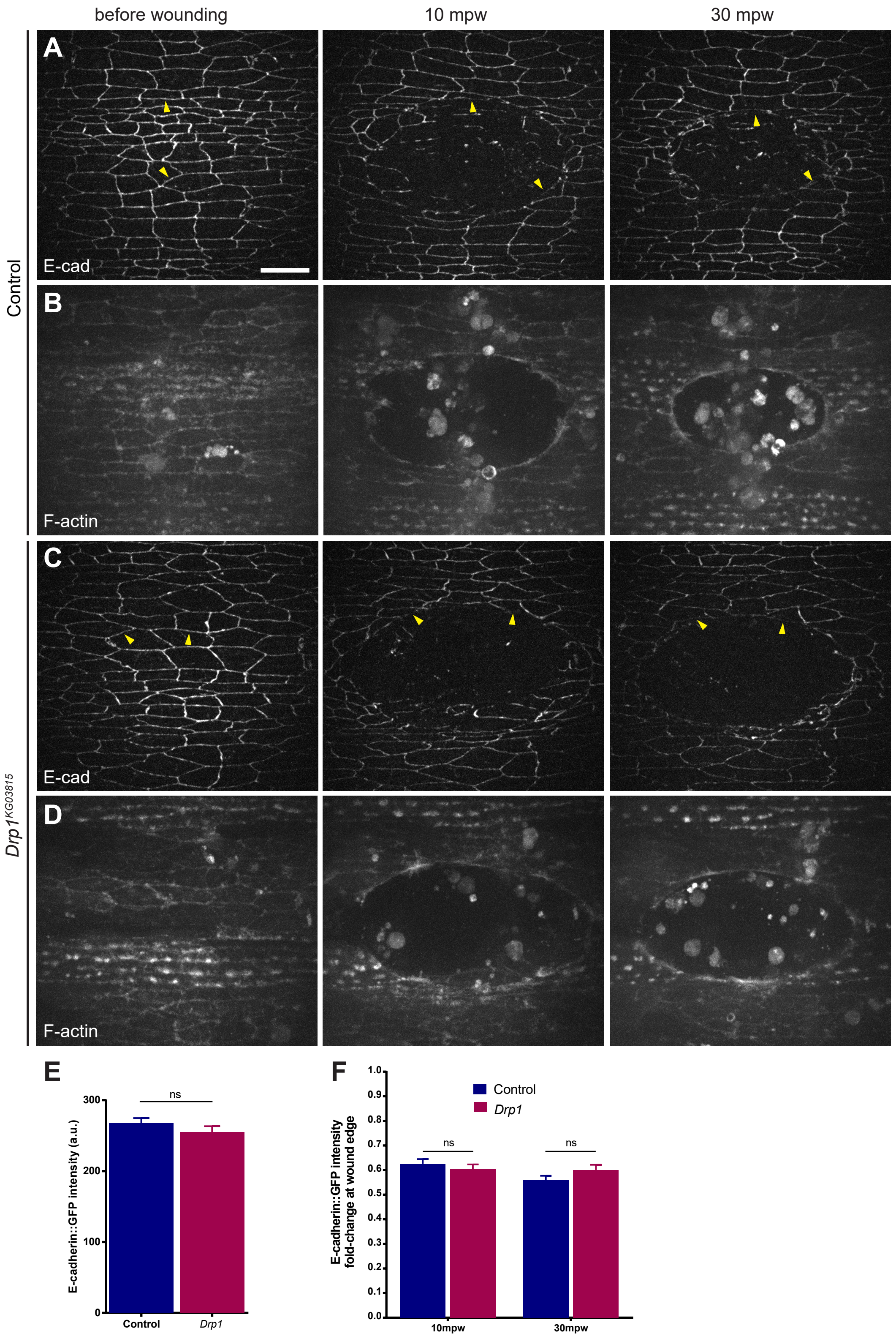

Biology Open • Supplementary information

Figure S2. E-cad localization in control and *Drp1* mutants. (A–D) Confocal images of the epidermis during wound closure in control (A and B) and *Drp1* mutants (C and D) expressing *ubi–E-cad::GFP* (A and C) and m*Cherry::Moesin* labeling F-actin (B and D) before and upon wounding. Upon wounding, E-cad intensity decreases at cell boundaries facing the wound edge in controls and *Drp1* mutants (arrowheads mark the same junctions before and after wounding in each embryo). Scale bar = 10 µm. **(E)** Graph of average E-cad fluorescence intensity in cells before wounding in control and *Drp1* mutant embryos. **(F)** Graph of fold change decrease in E-cad fluorescence intensity in cell boundaries at the wound edge at 10 and 30 mpw (compared with before wounding) in control and *Drp1* mutant embryos. N(control) = 52 junctions from 6 embryos; N(*Drp1*) = 64 junctions from 5 embryos. An unpaired *t* test (E) and a two-way ANOVA with a Tukey multiple comparisons test (F) were performed to test for significant differences between groups. Differences between groups are not significant (ns, $P > 0.05$). Error bars represent s.e.m.

Biology Open • Supplementary information

Figure S3

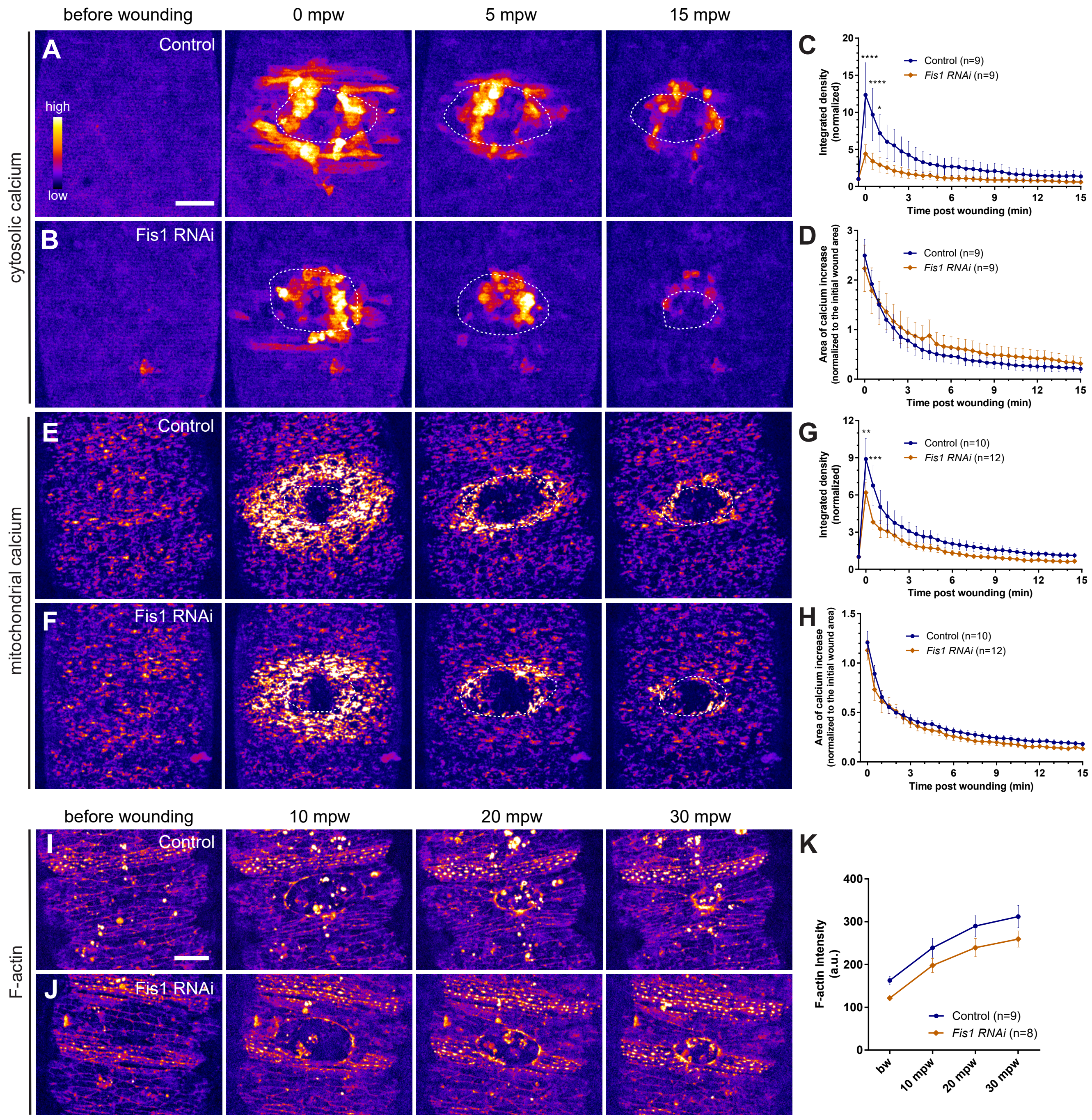

Biology Open • Supplementary information

Figure S3. Fis1 knockdown leads to altered cytosolic and mitochondrial Ca^{2+} dynamics upon wounding. (A, B) Maximum Z projections of the epidermis of control (A) and Fis1 RNAi (B) embryos expressing a cytosolic Ca^{2+} sensor (GCaMP6f) before and after wounding. Both control and Fis1 RNAi cells around the wound dramatically increase cytosolic Ca^{2+} levels immediately upon wounding (0 mpw). Ca^{2+} levels are lower in Fis1 RNAi (B, 0 mpw) embryos compared to controls (A, 0 mpw) but no difference was found in the area of cells that respond to the wound. **(C)** Graph of cytosolic Ca^{2+} intensity in control and Fis1 RNAi embryos shows that cytosolic Ca^{2+} is lower in Fis1 knockdown compared to controls in the first 1 mpw. **(D)** Graph of average area of elevated cytosolic Ca^{2+} in controls and Fis1 RNAi embryos shows that the Ca^{2+} burst area is similar to controls. **(E, F)** Maximum Z projections of the epidermis of control (E) and Fis1 RNAi (F) embryos expressing a mitochondrial Ca^{2+} sensor (mito::GCaMP3) before and after wounding. Wounding triggers an increase in mitochondrial Ca^{2+} levels in both control and Fis1 RNAi cells around the wound (E, F at 0 mpw). **(G)** Graph of mitochondrial Ca^{2+} intensity in control and Fis1 RNAi embryos. Fis1 RNAi embryos have a reduced Ca^{2+} burst in the first 0.5 mpw, compared to controls. **(H)** Graph of average area of elevated mitochondrial Ca^{2+} in controls and Fis1 RNAi embryos. No significant differences were found between control and Fis1 RNAi. Images are pseudo-colored with a gradient of fluorescence intensity, ranging from blue (low) to yellow (high). Dashed lines show the wound boundaries. A two-way ANOVA with a Sidak correction for multiple comparisons was used to test for significant differences between groups in C, D, G and F. Only significant differences are represented: * $P = 0.0379$, ** $P = 0.0034$, *** $P = 0.0009$, **** $P \leq 0.0001$. **(I-J)** Maximum Z projections of the epidermis of control (I) and *Fis1* RNAi (J) embryos expressing an F-actin (*mCherry::*Moesin) marker before and after wounding. Images are pseudo-colored with a gradient of fluorescence intensity, ranging from blue (low) to yellow (high). **(K)** Graph of average F-actin intensity at the cell cortex before wounding and at the wound edge. No differences were found in F-actin intensity between control and Fis1 RNAi embryos. A two-way ANOVA with a Sidak correction for multiple comparisons was used to test for significant differences between groups. Error bars represent s.e.m. Number of embryos per condition is shown in each graph. bw – before wounding. mpw – minutes post wounding. a.u. – arbitrary units. min – minutes. Scale bar = 20 µm.

Biology Open • Supplementary information

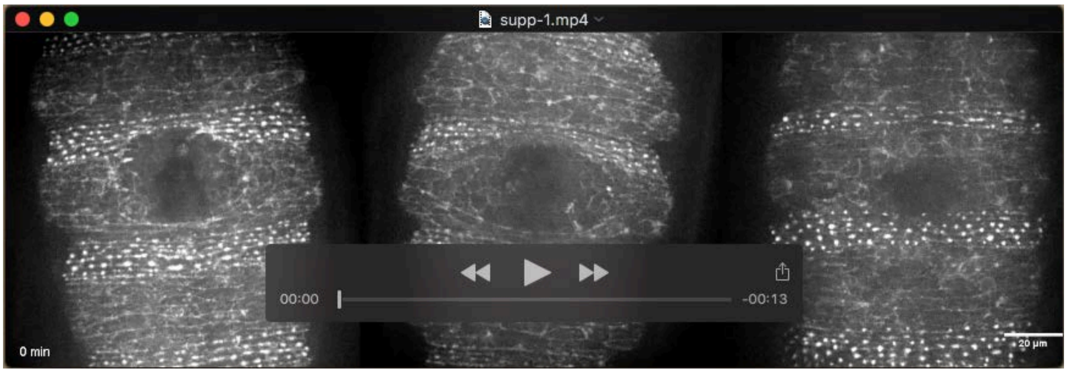

Movie 1. Wound closure dynamics in control, *Drp1* mild and *Drp1* strong embryos. Embryos express *GFP::Moesin* that marks F-actin. Images are maximum Z-projections of 55 slices (15.4-µm-thick stack) acquired by time-lapse spinning-disk confocal microscopy every 2 min. The frame rate is five frames/s. Video stills are shown in Fig. 2.

Biology Open • Supplementary information

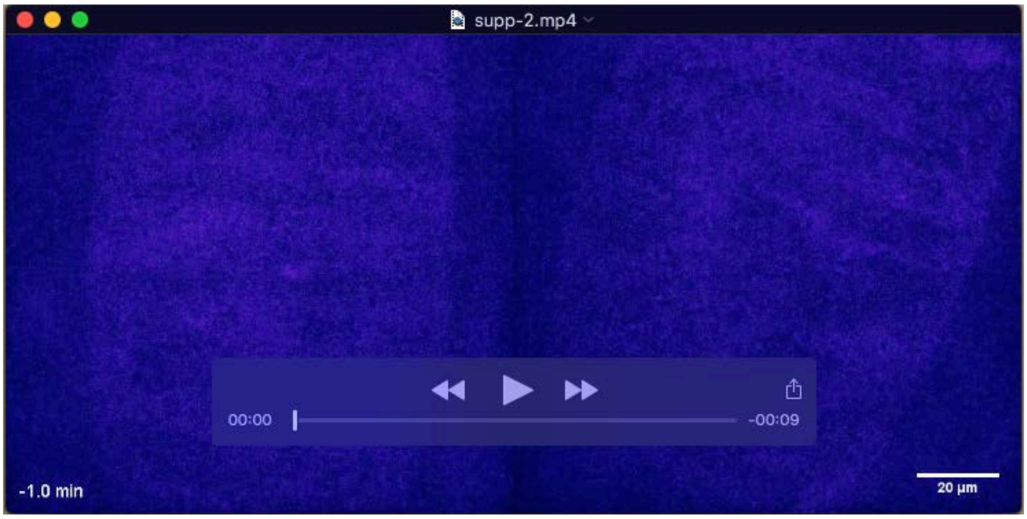

Movie 2. Cytosolic calcium dynamics in control and *Drp1* embryos during wound closure. Embryos express the cytosolic Ca^{2+} indicator *GCaMP6f*. Images are maximum Z-projections of 21 slices (10.4-µm-thick stack) acquired by time-lapse spinning-disk confocal microscopy every 30s and pseudo-colored in a gradient (ranging from lower intensities in blue to higher intensities in yellow). The frame rate is five frames/s. Video stills are shown in Fig. 4.

Biology Open • Supplementary information

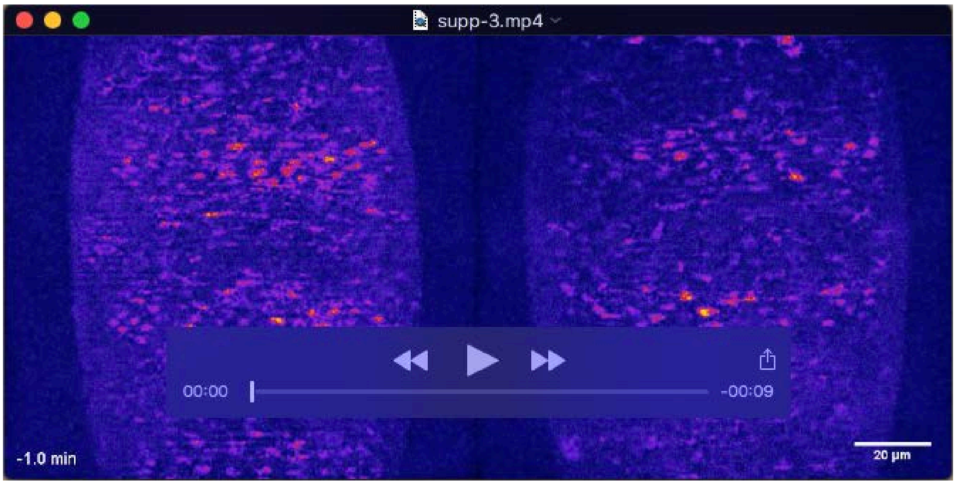

Movie 3. Mitochondrial calcium dynamics in control and *Drp1* embryos during wound closure. Embryos express the mitochondrial Ca^{2+} indicator mito::*GCaMP3*. Images are maximum Z-projections of 35 slices (17.5-µm-thick stack) acquired by time-lapse spinning-disk confocal microscopy every 30s and pseudo-colored in a gradient (ranging from lower intensities in blue to higher intensities in yellow). The frame rate is five frames/s. Video stills are shown in Fig. 4.
